# Role of Collagens and Perlecan in Microvascular Stability: Exploring the Mechanism of Capillary Vessel Damage by Snake Venom Metalloproteinases

**DOI:** 10.1371/journal.pone.0028017

**Published:** 2011-12-08

**Authors:** Teresa Escalante, Natalia Ortiz, Alexandra Rucavado, Eladio F. Sanchez, Michael Richardson, Jay W. Fox, José María Gutiérrez

**Affiliations:** 1 Instituto Clodomiro Picado, Facultad de Microbiología, Universidad de Costa Rica, San José, Costa Rica; 2 Departamento de Bioquímica, Escuela de Medicina, Universidad de Costa Rica, San José, Costa Rica; 3 Centro de Pesquisa e Desenvolvimento, Fundaçao Ezequiel Dias (FUNED), Belo Horizonte, Minas Gerais, Brazil; 4 Department of Microbiology, Immunology, and Cancer Biology, University of Virginia School of Medicine, Charlottesville, Virginia, United States of America; University of Giessen Lung Center, Germany

## Abstract

Hemorrhage is a clinically important manifestation of viperid snakebite envenomings, and is induced by snake venom metalloproteinases (SVMPs). Hemorrhagic and non-hemorrhagic SVMPs hydrolyze some basement membrane (BM) and associated extracellular matrix (ECM) proteins. Nevertheless, only hemorrhagic SVMPs are able to disrupt microvessels; the mechanisms behind this functional difference remain largely unknown. We compared the proteolytic activity of the hemorrhagic P-I SVMP BaP1, from the venom of *Bothrops asper*, and the non-hemorrhagic P-I SVMP leucurolysin-a (leuc-a), from the venom of *Bothrops leucurus,* on several substrates *in vitro* and *in vivo,* focusing on BM proteins. When incubated with Matrigel, a soluble extract of BM, both enzymes hydrolyzed laminin, nidogen and perlecan, albeit BaP1 did it at a faster rate. Type IV collagen was readily digested by BaP1 while leuc-a only induced a slight hydrolysis. Degradation of BM proteins *in vivo* was studied in mouse gastrocnemius muscle. Western blot analysis of muscle tissue homogenates showed a similar degradation of laminin chains by both enzymes, whereas nidogen was cleaved to a higher extent by BaP1, and perlecan and type IV collagen were readily digested by BaP1 but not by leuc-a. Immunohistochemistry of muscle tissue samples showed a decrease in the immunostaining of type IV collagen after injection of BaP1, but not by leuc-a. Proteomic analysis by LC/MS/MS of exudates collected from injected muscle revealed higher amounts of perlecan, and types VI and XV collagens, in exudates from BaP1-injected tissue. The differences in the hemorrhagic activity of these SVMPs could be explained by their variable ability to degrade key BM and associated ECM substrates *in vivo*, particularly perlecan and several non-fibrillar collagens, which play a mechanical stabilizing role in microvessel structure. These results underscore the key role played by these ECM components in the mechanical stability of microvessels.

## Introduction

Zinc-dependent metalloproteinases are abundant components in the proteomes of many snake venoms, especially in those of species of the family Viperidae [1], [2]. Snake venom metalloproteinases (SVMPs) are multi-domain proteins which have been classified in various classes on the basis of their domain composition [1]. Class P-I SVMPs comprise enzymes having, in their mature protein, only the metalloproteinase domain, whereas class P-II SVMPs present, in addition to the catalytic domain, a disintegrin domain, which may be cleaved to generate disintegrins. Enzymes of the P-III class have disintegrin-like and cysteine-rich domains following the metalloproteinase domain [1].

SVMPs play key roles in envenomations by snakes of the family Viperidae, and probably also in the case of some species of the family Colubridae (*sensu lato*) [3]–[7]. One of the most notorious effects of SVMPs is their ability to disrupt microvessels, provoking local and systemic hemorrhage [3], [8]. It has been proposed that this effect is the consequence of the hydrolysis of proteins forming the basement membrane (BM) of capillary blood vessels, a phenomenon that has been consistently demonstrated *in vitro* [9]–[17]. Although studies on BM damage *in vivo* have been scarce, a number of observations support the concept that capillary vessel BM is indeed affected by SVMPs when injected in tissues [16], [18]–[20]. A unified hypothesis, based on a two-step mechanism, has been proposed to explain the pathogenesis of hemorrhage by SVMPs [8], [21]. Initially, SVMPs hydrolyze key peptide bonds in BM and supporting extracellular matrix (ECM) components, promoting the weakening of the mechanical stability of BM. As a consequence, the hemodynamic biophysical forces normally operating in the vasculature, such as microvessel wall tension and shear stress, provoke the distention of the weakened capillary, which leads to microvessel disruption and extravasation [8].

Despite sharing a highly similar structure in their catalytic domain, SVMPs greatly differ in their capacity to induce hemorrhage [8], [22]. In general, P-III SVMPs are more potent hemorrhagic toxins than P-I SVMPs. This is likely to depend on the presence, in the former, of extra domains in addition to the catalytic one, since exosites in disintegrin-like and cysteine-rich domains enable these enzymes to bind to relevant targets in the extracellular matrix or in endothelial cells [1], [20], [23]–[26]. Moreover, P-III SVMPs are highly resistant to inhibition by the plasma proteinase inhibitor α_2_-macroglobulin (α_2_M), whereas P-I SVMPs are readily inhibited [27]–[32]. On the other hand, an intriguing observation is that a significant variation in hemorrhagic potency occurs also within the class P-I SVMPs [33], [34]. Since these enzymes comprise the metalloproteinase domain only, such difference in hemorrhagic activity depends on variations within this domain. Various proposals have been presented for identifying key structural determinants for hemorrhagic activity in P-I SVMPs [34]–[39], although this issue remains largely unsolved.

The functional differences between hemorrhagic and non-hemorrhagic P-I SVMPs have not been clarified either, as both groups are able to hydrolyze a variety of ECM components *in vitro* [11]–[13], [15]–[18], [22], [40], [41]. The cleavage patterns of several SVMPs on BM and plasma components *in vitro* have been investigated [12], [13], [16], [22], [42], but a systematic comparison between hemorrhagic and non-hemorrhagic enzymes, particularly regarding their ability to degrade BM and associated ECM components *in vivo,* is warranted. In addition to providing novel clues for understanding the pathogenesis of snake venom-induced hemorrhage, a highly relevant effect of viperid snakebite envenomations, this information will also shed light on the more general subject of the factors that determine the stability of microvessels, particularly regarding the mechanical supportive role played by BM and other ECM components in capillary vessels. The importance of this subject lies in the fact that many pathologies are associated with an impairment in the mechanical stability of the microvasculature.

In the present study we compared two P-I SVMPs having strikingly different hemorrhagic activity: BaP1 from the venom of *Bothrops asper*, and leucurolysin-a from *B. leucurus* venom. The comparison included their *in vitro* hydrolytic activity and the early *in vivo* degradation of BM and related ECM components. Results showed that hemorrhagic BaP1 induced a higher extent of degradation *in vivo* of several proteins that play a critical role in the mechanical stability of BM, thus supporting the concept that the hemorrhagic potential of SVMPs is related to the destabilizing consequences on BM structure of hydrolysis of selective ECM substrates by these enzymes.

## Methods

### Metalloproteinases

BaP1 and leucurolysin-a (leuc-a) were isolated from the venoms of *Bothrops asper* and *B. leucurus,* respectively, as previously described [38], [43], [44].

### Ethics statement

All *in vivo* experiments were performed in CD-1 mice (18–20 g). The experimental protocols involving the use of animals in this study were approved by the Institutional Committee for the Care and Use of Laboratory Animals (CICUA) of the University of Costa Rica (reference number 19–09).

### Quantification of hemorrhagic activity

Hemorrhagic activity was assessed by the quantification of hemoglobin present in muscle tissue, following a modification of the method used by Gutiérrez et al. [45]. Groups of four mice were injected intramuscularly (i.m.), in the right gastrocnemius, with either 50 µg of BaP1 or 50 µg of leuc-a, dissolved in 50 µL of 0.14 M NaCl, 0.04 M phosphate, pH 7.2 (PBS). Controls received 50 µL of PBS alone. After 15 min of injection, mice were sacrificed by CO_2_ inhalation and the muscles were dissected out. In order to extract the hemoglobin present in the muscle, samples were placed individually in tubes containing 1.5 mL of distilled water and incubated overnight at 4°C. Afterwards, an aliquot of the solution was withdrawn, centrifuged at 5000 rpm for 5 min, and diluted 1:2 with distilled water. Absorbance at 540 nm was measured as quantitative assessment of hemoglobin concentration.

### Proteolysis of azocasein and insulin B chain

Proteolytic activity of BaP1 and leuc-a on azocasein (Sigma–Aldrich) was assessed according to Wang et al. [46], with modifications. Briefly, various amounts of each SVMP, dissolved in 20 µL of 25 mM Tris, 150 mM NaCl, 5 mM CaCl_2_, pH 7.4, buffer were incubated with 100 µL of a 10 mg/mL solution of azocasein in the same buffer. After an incubation of 90 min at 37°C, the reaction was stopped by the addition of 200 µL of 5% trichloroacetic acid. Samples were then centrifuged at 2000 rpm, an aliquot of 150 µL of the supernatant was mixed with 150 µL of 0.5 M NaOH, and the absorbance at 450 nm was recorded. BaP1 cleavage bond specificity on oxidized insulin B-chain was assessed as previously described [44]. The substrate (0.75 mg) was dissolved in 0.675 mL of 20 mM Tris-HCl, pH 8.1, buffer and incubated with 0.4 µg BaP1 at 37°C at an enzyme:substrate (w/w) ratio of 1∶200. At various time intervals (1, 5, 15, 30 and 60 min), aliquots of 75 µL were withdrawn from the digestion mixture. The reaction was stopped by adding 10 µL glacial acetic acid, and samples were kept frozen until analysis. The peptides resulting from the digestion of the insulin B-chain were separated by HPLC in a column of Vydac C18 small pore (Vydac 2015P54) using a gradient of acetonitrile (0–60%, v/v) in aqueous 0.1% (v/v) trifluoroacetic acid during 60 min at a flow rate of 1 mL/min. The amino acid sequences of the purified peptides were determined using a Shimadzu PPSQ-21A protein sequencer. The positions cleaved by the enzyme were deduced by comparing the amino acid sequences with the known amino acid alignment of the insulin B-chain. The cleavage bond specificity of BaP1 on oxidized insulin B chain was compared with previously reported data for leuc-a [44].

### Inhibition of proteolytic activity by α_2_-macroglobulin (α_2_M)

Increasing amounts of α_2_M (2 to 16 µg) were incubated with a constant amount of BaP1 or leuc-a in a 500-µL final volume of 50 mM Hepes, pH 7.5, buffer. Molar ratios of enzyme: α_2_M of 0.27, 0.55, 1.1 and 2.2 were used. Samples were incubated at 37°C for 2 min and the proteinase activity was assayed on dimethylcasein as substrate, as described by Souza et al [47].

### Proteolysis of Matrigel

Matrigel (BD Biosciences) was used to assess the action of SVMPs on BM components. Matrigel is a solubilized BM-like composite from Engelbreth-Holm-Swarm sarcoma which is used as a surrogate of BM [48]. It is mainly composed of laminin, type IV collagen, nidogen and heparan sulphate proteoglycan. Matrigel was incubated with either BaP1 or leuc-a, at an enzyme:substrate ratio (w:w) of 1∶50. Incubations were performed for 15 min, 1 h and 3 h at 37°C. Reactions were stopped by the addition of 100 µL of RIPA buffer, containing 20 mM EDTA, and samples were frozen at −70°C. Matrigel incubated without SVMPs, under otherwise identical conditions and at the same time intervals, was used as control. In order to identify which substrates were cleaved preferentially by each SVMP, Matrigel mixtures were analyzed by Western blot using antibodies against laminin, nidogen, type IV collagen and perlecan. Briefly, 10 µg of each Matrigel mixture was separated on a 4–15% polyacrylamide gradient gel, under reducing or non-reducing conditions, transferred to a nitrocellulose membrane (Bio-Rad), and incubated with 2% low-fat milk in TBS. Some membranes were stained with Ponceau-S before blockade, to visualize transferred proteins. After blocking, membranes were incubated overnight at 4°C with one of the following antibodies: rabbit polyclonal anti-laminin at a dilution of 1∶1000 (Fitzgerald), rabbit polyclonal anti-type IV collagen at a dilution of 1∶2000 (Abcam), rabbit polyclonal anti-nidogen 1 at a dilution of 1∶6000 (Abcam), or rat monoclonal anti-perlecan at a dilution of 1∶300 (Millipore). Subsequently, membranes were incubated with either peroxidase-conjugated anti-rabbit IgG or peroxidase-conjugated anti-rat IgG (Jackson Immunoresearch), and the reaction was developed with a chemiluminescent substrate with the kit Novex (Invitrogen). Images were captured with ChemiDoc XRS+ (BioRad) and analysis was performed with ImageLab software (BioRad).

### Proteolysis of BM substrates *in vivo*

The effects of BaP1 and leuc-a on BM proteins *in vivo* were studied by injecting the enzymes, or PBS, in mouse gastrocnemius muscle. Groups of four mice were injected i.m., in the right gastrocnemius, with either 50 µg of BaP1 or 50 µg of leuc-a, dissolved in 50 µL of PBS. A control group injected with 50 µL of PBS alone was included. After 15 min of injection, mice were sacrificed by CO_2_ inhalation, muscles were resected and placed either in liquid nitrogen, for Western blot analysis, or in formalin free zinc fixative (BD Biosciences), for histology and immunohistochemistry.

### Western blot analysis of muscle homogenates

The muscles frozen in liquid nitrogen were pulverized in a mortar to the stage of fine particles. All muscles from each experimental treatment were pulverized together in order to prepare a pool. Each pool was resuspended in 1.5 mL of extraction buffer (8 M urea, 25 mM Tris-HCl, 150 mM NaCl, 1% Triton X-100, 0.1% SDS, 20 mM EDTA, pH 7.6) with a tablet of protease inhibitor cocktail (Roche) per 10 mL of buffer, and incubated for 2 h on ice with mild agitation. Samples were centrifuged and the supernatant was distributed in aliquots and stored at −70°C until analysis. Protein concentration was quantified with DC Protein Assay (BioRad). For immunoblotting, 40 µg protein of each sample were separated, under reducing or non-reducing conditions, on 4–15% Tris–HCl polyacrylamide gradient gels, and transferred to nitrocellulose membranes. Immunodetection was performed as described above, incubating the membranes with either rabbit polyclonal anti-laminin at a dilution of 1∶500 (Abcam), rabbit polyclonal anti-type IV collagen at a dilution of 1∶500 (Abcam), rabbit polyclonal anti-nidogen 1 at a dilution of 1∶8000 (Abcam), or goat polyclonal anti-endorepellin at a dilution of 1∶200 (R&D Systems). An anti-GAPDH antibody (Abcam), at a dilution of 1∶400, was used as loading control. The reaction was developed with a chemiluminescent substrate with the kit Novex (Invitrogen). Images were captured with ChemiDoc XRS+ (BioRad) and analysis was performed with ImageLab software (BioRad).

### Histology and immunohistochemistry

Resected muscles were placed in zinc fixative for 48 h, followed by routine processing and embedding in paraffin; then, 5 µm sections were mounted on positively charged slides (Thermo Scientific). For each sample, three transversal non-sequential sections were prepared. A set of slides was stained with hematoxylin-eosin for histological assessment of tissue alterations. Another set of slides was subjected to a double immunostaining, with anti-VEGFR-2, for endothelial cells, and anti-type IV collagen, for BM. Briefly, tissues were exposed to proteinase K for 3 min followed by blocking solution for 30 min. The tissue sections were then incubated with rat monoclonal anti-VEGFR-2 antibody (BD Biosciences), at a dilution of 1∶200. The binding was detected with a biotinylated anti-rat IgG (Dako), enhanced by a Tyramide signal amplification kit (Perkin Elmer), and visualized with streptavidin-Cy3 (Zymed Laboratories). The second immunolabeling was performed using rabbit polyclonal anti-type IV collagen at a dilution of 1∶500 (Fitzgerald Industries) followed by a biotinylated anti-rabbit IgG (Vector Laboratories). The remainder of the procedure was performed as described above and the reaction was visualized with streptavidin-Alexa 488 (Molecular Probes). Images were captured with a Cool SNAP-Pro camera (Media Cybernetics) and were analyzed with the software ImagePro Plus (Media Cybernetics). Capillary vessels were identified as round structures with a diameter of less than 10 µm and double stained with anti-VEGFR2 and anti-type IV collagen. In addition, skeletal muscle fibers were identified as cells of 40 µm of diameter or more, surrounded by type IV collagen immunostaining, and were quantified in the same images. On this basis, the capillary: muscle cell ratio was calculated.

### Proteomic analysis of wound exudates

To complement immunochemical techniques and to identify additional extracellular matrix substrates degraded by SVMPs, a proteomic analysis of the wound exudate induced by BaP1 or leuc-a was performed, as previously described [49]. Groups of four mice were injected in the right gastrocnemius with either 50 µg of BaP1 or 50 µg of leuc-a, dissolved in 50 µL PBS. After 15 min of injection, mice were sacrificed by CO_2_ inhalation, and a 5 mm incision was made with a scalpel in the skin overlying the injected muscle. Immediately, the sectioned skin was opened and a heparinized capillary tube was introduced under the skin to collect the wound fluid. An approximate volume of 20–50 ìL of exudate was collected from each mouse. Exudate samples were then pooled and lyophilized.

Lyophilized wound exudate samples were re-suspended in water and protein quantification was performed using NanoOrange protein kit (Invitrogen). Twenty micrograms of protein were acetone precipitated, resuspended in Laemmli buffer, applied to a 12% precast electrophoresis gel (Bio-Rad), separated, and stained with Coomassie Brilliant Blue. Gel lanes were cut in ten equal size slices. Gel slices were destained for 2 h and the proteins reduced (10 mM DTT) and alkylated (50 mM iodoacetamide) at room temperature. Gel slices were washed with 100 mM ammonium bicarbonate, dehydrated with acetonitrile and dried in a speed vac. Hydration of the slices was performed with a solution of Promega modified trypsin (20 ng/mL) in 50 mM ammonium bicarbonate for 30 min on ice. Excess trypsin solution was removed and the digestion was carried on for an additional 18 h at 37°C. Tryptic peptides were twice extracted from gel slices with 30 µL of a 50% acetonitrile/5% formic acid solution. The combined extracts were dried to a volume of 15 µL for mass spectrometric analysis. LC/MS/MS was performed using a Thermo Electron LTQ ion-trap mass spectrometer. Analytical columns were fabricated in-house by packing 7.5 cm Jupiter 10 µm C18 packing material (Phenomenex, Torrance, CA) into a 25 cm length of 360×75 µm fused silica (Polymicro Technologies, Phoenix, AZ) behind a bottleneck. Samples were loaded directly onto these columns for the C18 analytical runs. In-gel digests (50% of each sample) were injected into the mass spectrometer at 300 nL/min. Peptides were eluted from the C18 column using an acetonitrile/0.1M acetic acid gradient (2–90% acetonitrile). The instrument was programmed to acquire a cycle of one mass spectrum followed by MS/MS on the ten most abundant ions in a data-dependent mode. After MS/MS, fragmentation was carried out on a particular parent ion and the m/z was placed on an exclusion list for 2 min to enable greater dynamic range and prevent repeat analysis of the same ions. The electrospray voltage was set to 2.5 kV, and the capillary temperature was 230°C.

The mass spectra were extracted and analyzed utilizing Bioworks Sequest 3.11 software. Searches were performed against a mouse IPI nonredundant database (http://www.ebi.ac.uk/IPI/). Spectra generated on the LTQ were searched using 1.5 Da parent tolerance and 1 Da fragment tolerance. All hits were required to be fully tryptic. The results from the searches were exported to Scaffold (version 2.2.03, Proteome Software Inc., Portland, OR). Scaffold was used to validate MS/MS based peptide and protein identifications and to visualize multiple datasets in a comprehensive manner. Confidence of protein identification in Scaffold is displayed as a Probability Legend with green coloration indicative of over 95% confidence and yellow as 80% to 94% confidence. Relative quantization of proteins was accomplished by summing all data from the 10 gel slices for a particular sample in Scaffold and then displaying the Quantitative Value from the program. This number gives an average total of non-grouped spectral counts for a protein divided by the total non-grouping spectral counts for the 10 mass spectral runs from the gels slices from each lane (http://www.proteomesoftware.com/). This format of presentation allows for a relative quantitative comparison between a specific protein from different samples and to a certain degree gives some measure of relative abundance between proteins generated from the mass spectrometric analysis of the 10 gel slices for a particular exudate sample. Some of the data were further analyzed manually to determine if mass spectra were derived from proteins migrating in the gel at their expected molecular mass or at a lower mass.

### Statistical analysis

To assess the statistical significance of the differences in the mean values of experimental groups, an analysis of variance was performed, followed by a Tukey–Kramer test to compare pairs of means. P values <0.05 were considered significant.

## Results

### Hemorrhagic activity

**Figure 1:**
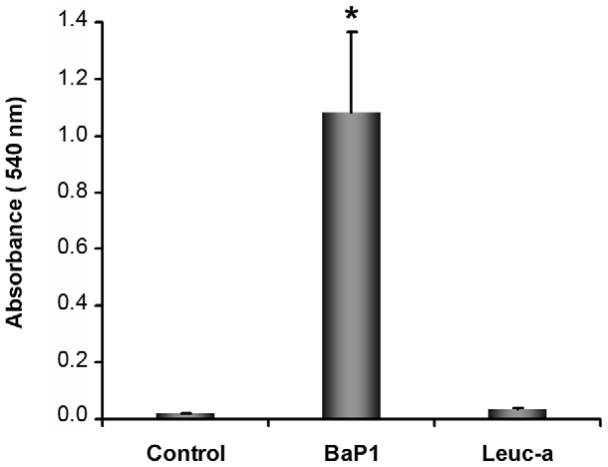
Hemorrhagic activity of BaP1 and leuc-a in mouse muscle. Mice were injected intramuscularly, in the gastrocnemius, with 50 µg of either BaP1 or leuc-a, dissolved in 50 µL PBS, or with 50 µL PBS (controls). After 15 min, mice were sacrificed and muscles were dissected out and placed in 1.5 mL of distilled water. Samples were incubated overnight at 4°C and the absorbance at 540 nm was recorded in the supernatant as an indicator of muscle hemoglobin content. BaP1 induced a profuse hemorrhage whereas leuc-a did not exert hemorrhagic activity. Results are presented as mean ± SD (n = 4). *P<0.05 when compared to control and leuc-a treatments.

At the dose of 50 µg, BaP1 induced a conspicuous hemorrhage in the gastrocnemius muscle of mice after 15 min of injection (Fig 1). In contrast, the same amount of leuc-a did not induce hemorrhage, although there was conspicuous edema in the tissue. Muscle injected with PBS did not show hemorrhage (Fig 1) or edema.

### Proteolysis of azocasein and insulin B chain, and inhibition by α_2_M

**Figure 2:**
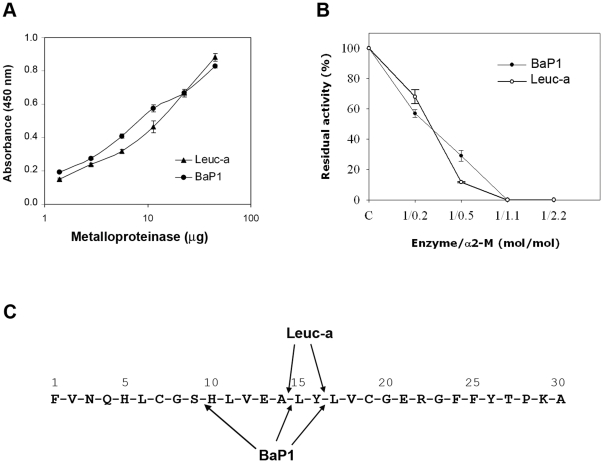
Proteolytic activity of BaP1 and leuc-a, and inhibition by α_2_-macroglobulin. (**A**) Hydrolytic activity of BaP1 and leuc-a on azocasein. Various amounts of each enzyme were incubated with azocasein for 90 min at 37°C. The reaction was stopped by the addition of 5% trichloroacetic acid, and the absorbances of the supernatants at 450 nm were recorded after centrifugation. Controls of azocasein without enzyme were run in parallel and their absorbance was subtracted from the sample values. Results are presented as mean ± S.D. (n = 3). (**B**) Stoichiometry of inhibition of BaP1 and leuc-a by α_2_M. The plasma inhibitor was incubated with BaP1 or leuc-a at various molar ratios, and proteolytic activity was tested on dimethylcasein. The remaining protease activity is expressed as percentage of the original activity measured in the absence of the inhibitor. Results are presented as mean ± SD (n = 3). (**C**) Cleavage sites of BaP1 and leuc-a on oxidized insulin B-chain. After 30 min of digestion, peptides were separated by HPLC and identified by their amino acid sequence.

Proteolytic activity on azocasein was similar for BaP1 and leuc-a (Fig 2A). Proteolytic activity of both enzymes was similarly inhibited by α_2_M, with complete inhibition observed at an enzyme/inhibitor molar ratio of 1∶1.1 (Fig 2B). Both SVMPs cleaved oxidized insulin B chain at positions Ala_14_-Leu_15_ and Tyr_16_-Leu_17_; additionally, BaP1 cleaved the Ser_9_-His_10_ bond (Fig 2C).

### Proteolysis of Matrigel

**Figure 3:**
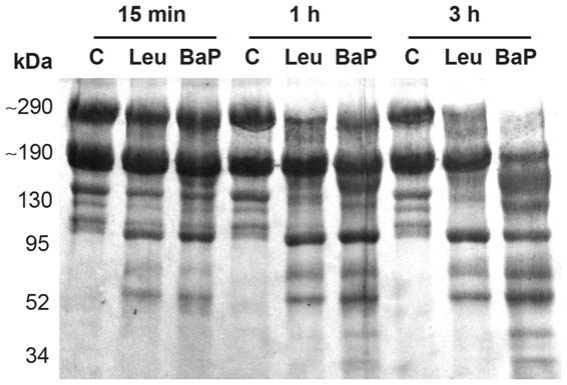
Hydrolysis of Matrigel proteins by BaP1 and leuc-a. Matrigel was incubated at 37°C with each enzyme at a 1∶50 (w:w) enzyme:substrate ratio for 15 min, 1 h and 3 h. A control of Matrigel without enzymes (C) was included for each time interval. Matrigel solutions were separated by SDS–PAGE under reducing conditions using a 4–15% gradient gel, and transferred to nitrocellulose membrane and stained with Ponceau-S.

When tested on Matrigel, at various incubation times, both enzymes degraded the predominant bands, with the appearance of degradation products observed within 15 min of incubation (Fig 3). At this time, BaP1 and leuc-a generated fragments of 100, 75 and 60 kDa. A higher extent of hydrolysis was observed for BaP1, compared to leuc-a, at 1 and 3 h, with the observation of additional proteolytic fragments of 155, 130, 40 and 30 kDa, and a higher reduction in the intensity of the bands of ∼300 and ∼ 200 kDa (Fig 3).

### Western blot analysis of BM degradation *in vitro* and *in vivo*

#### Laminin

**Figure 4:**
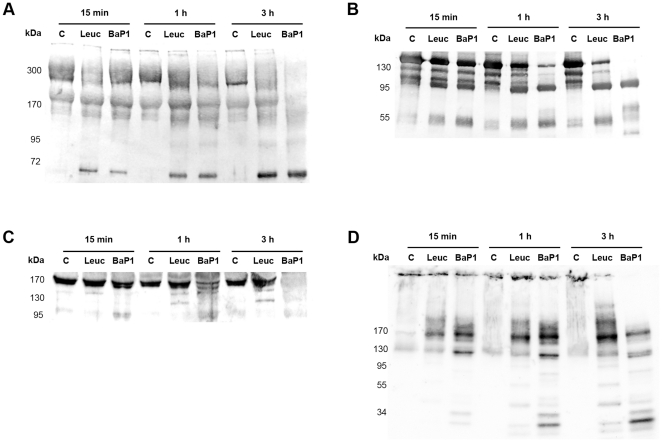
Hydrolysis of basement membrane components *in vitro*. Hydrolysis of laminin (A), nidogen (B), type IV collagen (C) and perlecan (D) by BaP1 and leuc-a, as detected by Western blotting of Matrigel. Matrigel was incubated with either BaP1, leuc-a or PBS (control, lane C), as described in the legend of Fig 3. Matrigel preparations were separated by SDS-PAGE under reducing conditions using a 4-15% gradient gel and transferred to nitrocellulose membranes. Immunodetection was performed with either anti-laminin, anti-nidogen, anti-type IV collagen or anti-perlecan antibodies. Reaction was developed with a chemiluminiscent substrate.

**Figure 5:**
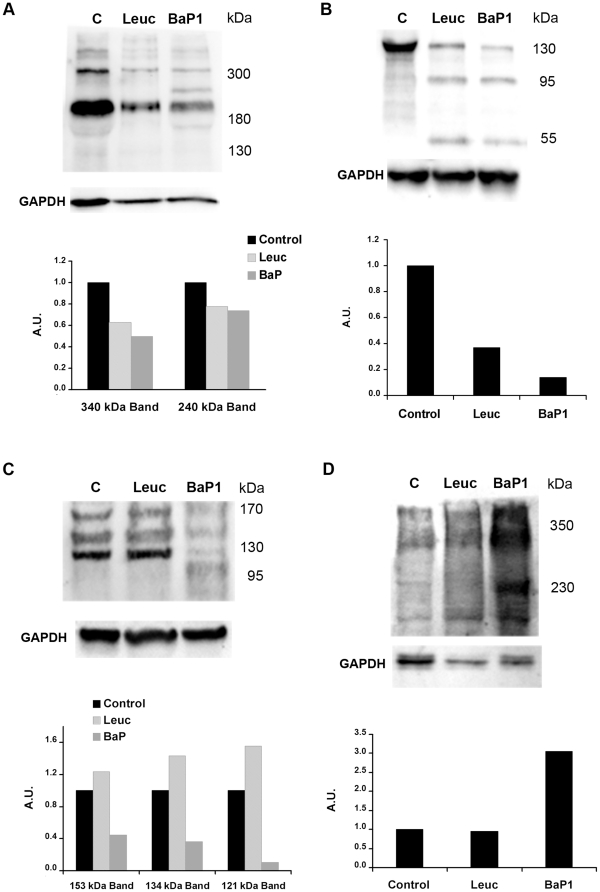
Hydrolysis of basement membrane components *in vivo*. Hydrolysis of laminin (A), nidogen (B), type IV collagen (C) and perlecan (D) by BaP1 and leuc-a, as detected by Western blotting of homogenates of injected mouse gastrocnemius muscle. Groups of mice were injected in the gastrocnemius muscle with either 50 µg BaP1, 50 µg leuc-a or PBS (lane C). After 15 min, mice were sacrificed and tissue was homogenized and centrifuged to obtain the supernatant. Supernatants of muscle homogenates were separated by SDS-PAGE under reducing conditions, using a 4–15% gradient gel, and transferred to nitrocellulose membranes. Immunodetection was performed with either anti-laminin, anti-nidogen, anti-type IV collagen or anti-endorepellin antibodies, and with anti-GAPDH as loading control in tissue homogenates. Reaction was developed with a chemiluminiscent substrate. Densitometry was carried out in blots of tissue homogenates with ImageLab software; a relative quantification was performed adjusting each sample to the corresponding control.

Immunodetection of laminin in Matrigel samples by Western blot identified two main bands of ∼310 and 200 kDa in control samples, which are likely to correspond to laminin chains α1 and β1, respectively. BaP1 and leuc-a hydrolyzed laminin chains, generating fragments of 150, 95 and 60 kDa after 15 min of incubation (Fig 4A; Table 1). Nevertheless, BaP1 cleaved laminin more readily than leuc-a and caused a more prominent reduction of the 310 and 200 kDa bands at 1 and 3 h (Fig 4A). In muscle homogenates from control mice, anti-laminin antibodies recognized four main bands of ∼400, ∼340, 240 and 180 kDa. Homogenates from tissue injected with either BaP1 or leuc-a presented a similar reduction in the intensity of these bands as compared with controls. In addition, BaP1 induced the appearance of a degradation product of 285 kDa. (Fig 5A; Table 1).

**Table 1:** Mol. mass of most abundant fragments of BM proteins generated by BaP1 and Leuc-a.

	Matrigel *in vitro*		*In vivo*	
Protein	Leuc-a	BaP1	Leuc-a	BaP1
Type IV Collagen	140, 120	155, 140, 100	-a	110
Laminin	150, 95, 60	150, 95, 60	-b	285
Nidogen	95, 50	95, 50, 40	100, 50	100, 50
Perlecan	170, 120, 40,30	170, 120, 35, 30	-c	230

a No degradation bands were detected by immunoblotting, and the intensity of the main bands of type IV collagen was not reduced.

b No degradation bands were detected by immunoblotting, but the main laminin bands were reduced as compared to control samples.

c No degradation bands were detected by immunoblotting.

#### Nidogen

Western blot analysis of Matrigel samples showed a rapid degradation of the 150 kDa nidogen band by both SVMPs, with the generation of fragments of 95 and 50 kDa after 15 min of incubation; however, proteolysis by BaP1 occurred at a faster rate (Fig 4B; Table 1). A 40 kDa degradation product was generated by BaP1, but not by leuc-a, at 3 h of incubation (Fig 4B; Table 1). Immunodetection of nidogen in muscle samples from mice treated with both SVMPs clearly showed hydrolysis of this protein *in vivo* as early as 15 min of injection of the toxins, with the appearance of degradation products of 100 and 50 kDa (Fig 5B; Table 1). There was a higher extent of hydrolysis by BaP1 than by leuc-a (Fig 5B).

#### Type IV collagen

Immunoblotting of Matrigel with anti-type IV collagen antibodies revealed a 170 kDa band in control and SVMP-treated samples (Fig 4C). Degradation of this band by BaP1 was evident within 15 min of incubation and increased with longer incubation times. In contrast, leuc-a treatment induced only a slight reduction of the 170 kDa band after 3 h of incubation (Fig 4C). Different proteolytic fragments were generated by the two SVMPs: 140 and 120 kDa fragments by leuc-a, and 155, 140 and 100 kDa by BaP1, indicative of different cleavage sites (Fig 4C; Table 1). Western blot of control muscle homogenates detected three main bands of 155, 135 and 120 kDa (Fig 5C; Table 1). All immunodetected bands were significantly reduced only by BaP1 treatment, and a degradation fragment of 110 kDa was detected (Fig 5C; Table 1).

#### Perlecan

Control samples of Matrigel incubated without SVMPs showed a high molecular mass band (arrow in Fig 4D). Several fragments of lower molecular mass were detected in Matrigel samples incubated with either leuc-a or BaP1, demonstrating that both enzymes can hydrolyze this BM component *in vitro*, generating a similar pattern of degradation. BaP1 generated fragments of 170, 120, 35 and 30 kDa, whereas leuc-a generated fragments of 170, 120, 40 and 30 kDa (Fig 4D; Table 1). However, the rate of degradation was faster with BaP1 since, at 15 min and 1 h, the intensity of the bands corresponding to degradation products was higher than in samples from leuc-a-treated mice (Fig 4D). The monoclonal antibody against perlecan did not yield satisfactory results when tested on muscle homogenates. Therefore, a polyclonal antibody against endorepellin, the C-terminal domain of perlecan, was used for detection of the proteoglycan *in vivo*. Immunoblotting with this antibody demonstrated the presence of a diffuse band of approximately 350 kDa in all samples analyzed, being more pronounced in samples from muscle injected with BaP1 (Fig 5D). Moreover, in samples from BaP1-injected mice, a 230 kDa band not present in control or leuc-a samples was detected (Fig 5D; Table 1).

### Histology and immunohistochemistry

**Figure 6:**
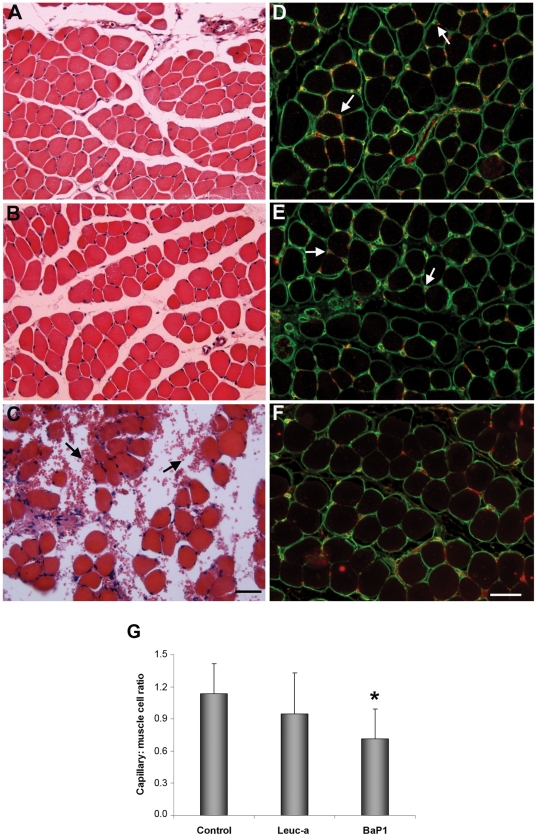
Histological and immunohistochemical analysis of the effects of BaP1 and leuc-a in skeletal muscle. Mice were injected with either 50 µL PBS as control (A, D), 50 µg of leuc-a (B, E) or 50 µg BaP1 (C, F). Tissue samples were collected 15 min after injection and processed for embedding in paraffin (see experimental details in Methods). A, B and C: Hematoxylin-eosin staining. Hemorrhage, evidenced by the presence of erythrocytes in the interstitial space (arrows), occurred only in muscle injected with BaP1. D, E and F: Immunostaining with anti-type IV collagen (green) and anti-VEGFR2 (red). Arrows depict capillary vessels. There is a reduction in capillary vessels positive for type IV collagen and VEGFR2 in samples treated with BaP1, whereas no reduction in the staining of capillaries was observed in tissue injected with leuc-a. Bar corresponds to 50 µm. (G) The total number of capillaries and muscle cells were counted in various sections, and the capillary: muscle cell ratio was calculated. Results are presented as mean ± SD. A significant reduction in the ratio was observed only in muscle injected with BaP1, but not with leuc-a. *P<0.05 when compared with capillary: muscle cell ratios of control and leuc-a-treated samples.

Muscle sections from control mice injected with PBS alone showed a normal tissue structure with bundles of muscle fibers surrounded by connective tissue (Fig 6A). Tissue sections from mice injected with leuc-a showed a morphology similar to controls, and there was no evidence of erythrocytes in the endomysium or perimysium (Fig 6B). In contrast, BaP1-treated samples presented hemorrhage, with masses of erythrocytes in the interstitial space surrounding muscle fibers (Fig 6C).

Since type IV collagen was the substrate which showed the most significant differences in the degradation patterns between the two SVMPs, detection of this protein by immunohistochemistry was performed. Immunostaining for type IV collagen and VEGFR-2, an endothelial cell marker, in sections of muscle injected with PBS showed a normal distribution of capillaries around the muscle fibers (Fig 6D). Sections from tissue injected with leuc-a showed a pattern of immunostaining of these markers which was similar to control samples (Fig 6E). In contrast, BaP1 induced a reduction in the immunostaining for type IV collagen and VEGFR-2 in capillaries (Fig 6F). The capillary : muscle cell ratio in muscle was estimated after the quantification, in tissue sections, of the numbers of capillaries and muscle cells. The capillary : muscle cell ratio in muscle injected with BaP1 was 0.7±0.3, which is significantly lower (p<0.05) than in control muscle injected with PBS (Fig 6G), whereas the capillary : muscle cell ratio in leuc-a treated muscle did not show a significant drop, as compared with PBS-treated muscle (p>0.05) (Fig 6G), in agreement with the lack of hemorrhagic activity of this SVMP.

### Proteomic analysis of wound exudates

**Figure 7:**
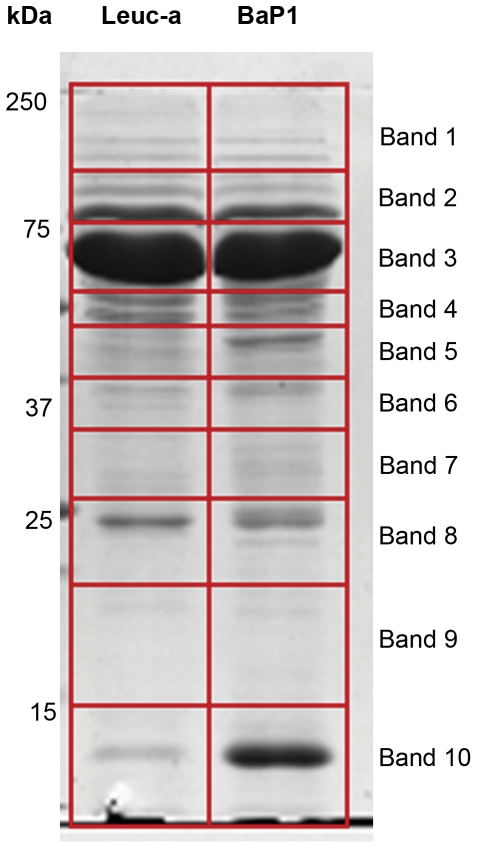
SDS-PAGE of exudates samples collected from mice injected with either BaP1 or leuc-a. Samples corresponding to 20 µg protein of exudates collected 15 min after injection of SVMPs were electrophoresed on a 4–20% gradient gel followed by staining with Coomassie Blue. Molecular mass markers are depicted to the left. Gel lanes were cut into ten equal size slices for further proteomic analysis (see Methods for details).

The electrophoretic profile of wound exudates collected from mice injected with BaP1 and leuc-a is shown in Fig 7. Similar profiles were observed with both enzymes, although exudate collected from BaP1-injected mice showed a higher intensity in a low molecular mass band which very likely corresponds to hemoglobin, according to proteomic analysis (not shown), in agreement with the hemorrhagic activity of this SVMP. From the mass spectrometric analysis of the gel bands of these exudates, more than 400 proteins were identified with XCorr Scores equal of greater than 1.8, 2.3, 2.7 and 3.7 for +1, +2, +3 and +4 ions, respectively, and protein identification probability above 95%. Extracellular matrix proteins detected in the wound exudates are listed in Table 2. Of the most abundant extracellular matrix proteins identified, 9 showed differential abundances of greater than three-fold between the two SVMPs. Of these, the fibrillar type III collagen was more abundant in the exudate from leuc-a-injected tissue. In contrast, the non-fibrillar types VI and XV collagens, together with perlecan, were present in higher amounts in exudates collected from mice injected with BaP1. No marked differences were observed in laminin and nidogen between treatments (Table 2).

**Table 2:** Proteomic analysis of extracellular matrix proteins in exudates collected from mice injected with BaP1 or Leuc-a.

			Quantitative Value		
Protein	Accession #	Mol. Weight	BaP1	Leuc-a	Fold change
Basement membrane-specific heparan sulfate proteoglycan core protein	IPI00113824	398 kDa	24 *	6	4.0
Biglycan	IPI00123194	42 kDa	2	1	2.0
Collagen I alpha-1 chain (isoform 1)	IPI00329872	138 kDa	20	42	2.1
Collagen I alpha-2 chain	IPI00222188	130 kDa	19	32	1.7
Collagen III alpha-1 chain	IPI00129571	139 kDa	8	25	3.1
Collagen VI alpha 3 chain	IPI00830749	289 kDa	39	9	4.3
Collagen VI alpha 3 subunit	IPI00845618	164 kDa	0	6	6.0
Collagen VI alpha3 (Fragment)	IPI00877197	186 kDa	3	0	>3
Collagen VII alpha-1 chain	IPI00134652	295 kDa	1	1	1.0
Collagen XI alpha-2 chain (isoform 7)	IPI00138069	162 kDa	1	1	1.0
Collagen XII alpha-1 chain (isoform 1)	IPI00121430	340 kDa	10	8	1.3
Collagen XIV alpha-1 chain (isoform 1)	IPI00330632	193 kDa	24	19	1.3
Collagen XV alpha-1 chain	IPI00409035	140 kDa	9	0	>9
Collagen XVIII alpha-1 chain (isoform 2)	IPI00131476	156 kDa	2	3	1.5
Decorin	IPI00123196	40 kDa	7	6	1.2
EGF-containing fibulin-like extracellular matrix protein 1	IPI00223457	55 kDa	3	10	3.3
Fibronectin	IPI00113539	272 kDa	347	365	1.1
Fibulin-1 (isoform C)	IPI00230432	75 kDa	3	2	1.5
Laminin subunit alpha-1	IPI00113726	338 kDa	1	2	2.0
Laminin subunit beta-3	IPI00117093	129 kDa	2	1	2.0
Laminin, gamma 2	IPI00117115	130 kDa	2	2	1.0
Lumican	IPI00313900	38 kDa	47	44	1.1
Nidogen-1	IPI00111793	137 kDa	11	18	1.6
Nidogen-2	IPI00129903	154 kDa	1	2	2.0
Perlecan	IPI00515360	470 kDa	3	0	>3
Tenascin (isoform 1)	IPI00403938	232 kDa	2	1	2.0
Tenascin X	IPI00130794	435 kDa	22	12	1.8
Thrombospondin-1	IPI00118413	130 kDa	20	1	20.0
Vitronectin	IPI00129240	55 kDa	41	70	1.7

*Values underlined correspond to proteins whose quantitative value differed by more than 3 fold when comparing the two SVMPs.

## Discussion

BM and associated ECM play a key role in the mechanical stability of capillary blood vessels [50]. SVMPs constitute relevant tools to explore the relevance of these ECM components in the microvasculature, owing to the differential ability of these venom components to damage capillary vessels. BaP1 and leuc-a are P-I SVMPs which have a high sequence identity, and similar 3D structure [34], enzymatic activity on azocasein *in vitro*, cleavage pattern on insulin B chain, and inhibition by α_2_M. Moreover, both have a clear preference for leucine in the P1′ site [42]. Despite these extensive similarities, BaP1 exerts hemorrhagic activity in mice whereas leuc-a is unable to induce bleeding even when injected at high doses. Our findings show that, in contrast with their similar proteolytic activity on azocasein, these enzymes differ in their capacity to hydrolyze BM proteins *in vitro* and, more relevantly, *in vivo.* This observation may be central to their variable hemorrhagic potential. Degradation of Matrigel revealed a similar pattern of hydrolysis on laminin, nidogen and perlecan, although BaP1 degraded these proteins at a faster rate than leuc-a. Despite the fact that these observations correspond to *in vitro* experiments, which need to be extrapolated with caution, they reveal a time-dependent difference in the ability of these SVMPs to degrade BM components, a finding that may be relevant *in vivo* from the toxicokinetic standpoint. Once injected in the tissue, toxins diffuse and are reabsorbed by several mechanisms, including lymphatic drainage and, therefore, have a time frame within which they can cleave BM components to induce hemorrhage. On the other hand, a more interesting difference between these SVMPs was observed in terms of hydrolysis of type IV collagen in Matrigel. BaP1 readily cleaved this BM component, whereas leuc-a hydrolyzed it to a much lesser extent. Earlier investigations demonstrated *in vitro* degradation of type IV collagen by SVMPs [11], [12], [15], although these studies did not evidence striking differences between SVMPs in their ability to degrade this substrate.

Many works have analyzed the hydrolysis of BM components by SVMPs *in vitro*. However, information on BM degradation *in vivo* is scarce; immunohistochemical data have demonstrated the ability of hemorrhagic SVMPs to degrade BM components in mice [16], [20]. Our present observations highlight marked differences in the ability of hemorrhagic and non-hemorrhagic P-I SVMPs to hydrolyze type IV collagen and perlecan in tissue homogenates of mouse gastrocnemius muscles injected with these enzymes. In agreement, exudate analysis also revealed higher amounts of perlecan in samples collected from mice injected with BaP1 than in those receiving leuc-a. To the best of our knowledge, this is the first report of degradation of perlecan by SVMPs both *in vitro* and *in vivo*. Our results also demonstrate that BM proteins can be readily hydrolyzed by SVMPs *in vivo* within a short time span, in accordance with the rapid onset of hemorrhage induced by these enzymes [18], [19], [21]. Of particular relevance is the observed degradation of type IV collagen *in vivo,* since BaP1 readily cleaved this protein within 15 min of injection, whereas no apparent digestion was observed in the case of leuc-a. These observations are relevant in the light of the role played by type IV collagen, perlecan and nidogen in the scaffolding and mechanical stability of capillary BM [50].

Current views on the assembly and structure of BM indicate that the initial polymeric scaffold of BM is provided by laminin, which is also responsible for early cell attachment of immature BMs. Assembly is then completed with the integration of a network of type IV collagen. Subsequently, other BM components, such as nidogen, perlecan and other proteins are incorporated and provide mechanical stability and complexity to the BM scaffold [50]–[52]. Type IV collagen plays a key role in BM assembly and stability, as it constitutes the only covalently-stabilized network polymer in BM [50], thus greatly contributing to the mechanical stability of this extracellular matrix structure. It has been demonstrated that mutations on type IV collagen genes cause structural deficiencies in capillary BM that lead to hemorrhage in mouse embryos and brain hemorrhage in humans [53]–[55]. In addition, alveolar and glomerular hemorrhage is a common manifestation in autoimmune diseases associated with anti-type IV collagen autoantibodies [56]. Perlecan interacts with other BM components, thus contributing to the stability and mechanical integration of BMs [50], [57]–[60]. Homozygous mice with a null mutation in the perlecan gene present deterioration of BM in regions of increased mechanical stress, such as the contracting myocardium, and often develop hemorrhage in the pericardial cavity [61]. Nidogen also constitutes a key basal lamina protein, acting as an integrating element that binds to laminin and type IV collagen networks [51] and is also relevant for capillary basal lamina stability [62].

In this context, the higher degradation *in vivo* of nidogen and perlecan, and especially of type IV collagen, by BaP1 is likely to explain its ability to induce hemorrhage. On the other hand, the fact that leuc-a treated muscles also present degradation of nidogen in the absence of hemorrhage suggests that hydrolysis of this protein alone is not sufficient to destabilize the BM, and that a concomitant hydrolysis of other components is required for hemorrhage to occur. Likewise, the similar hydrolytic activity of these SVMPs on laminin is compatible with the concept that the main role of this BM component is related to the formation of BM initial scaffold and to the interaction with plasma membrane-associated proteins rather than a mechanically-stabilizing role [50], [52]. On the basis of our findings, it is suggested that the ability to cleave type IV collagen is critical for the hemorrhagic action of P-I SVMPs. The relevance of binding and hydrolysis of type IV collagen in the action of hemorrhagic SVMPs has been also shown for the P-III SVMP jararhagin [20], [26], [63] and for a hemorrhagic metalloproteinase from the prokaryote *Vibrio vulnificus* [64]. Our immunohistochemical results provide further insights into the mechanism of SVMP-induced capillary damage. An evident loss of immunostaining for type IV collagen and VEGFR-2, i.e. endothelial cells, was observed in muscle tissue injected with BaP1. In the case of leuc-a, no loss of type IV collagen immunostaining was observed, in agreement with its lack of hemorrhagic activity.

Proteomic analysis of wound exudates constitutes a valuable tool to detect pathological alterations in tissues [49], [65]. Fragments of BM components have been detected by immunoblotting in exudates from mice injected with BaP1 [66]. The proteomic analysis of wound exudates performed in this study provides a broader view on the ability of these SVMPs to affect extracellular matrix components, including those of BMs. Exudates collected from mice injected with BaP1 had higher amounts of perlecan, and types VI and XV collagens. Type VI collagen plays a role of connecting BM components with fibrillar collagens of the matrix [67], [68]. It interacts with type IV collagen [68] as well as with perlecan and fibronectin [69]. Congenital deficiencies in type VI collagen, such as in Ullrich's disease, are associated with myopathy and with structural alterations in capillaries, which show replication of BM, an observation suggestive of microvessel damage [70]. Type XV collagen is a heparan sulphate proteoglycan often expressed in BM zones, i.e. a region contiguous to BM where various proteins anchor BM to subjacent connective tissue, and is likely to function also as a structural BM organizer [60]. Therefore, types VI and XV collagens also play a relevant role in the mechanical stability of BMs and on the integration of BM with subjacent connective tissue. The presence of relatively higher amounts of degradation products of these proteins, as well as of perlecan, in exudate from mice injected with BaP1, as compared with leuc-a, strongly suggests that BaP1 has a higher capacity to hydrolyze these critical structural components, and this may have implications in the pathogenesis of microvessel damage leading to hemorrhage. The role of hydrolysis of type VI collagen and other FACITs in the mechanism of action of hemorrhagic SVMPs has been previously proposed [24], [49], [71].

The absence of type IV collagen in the exudates is noteworthy, since immunochemical analyses of muscle homogenates revealed a conspicuous hydrolysis of this component. This discrepancy might be explained by the fact that type IV collagen network is stabilized by covalent bonding; thus, upon cleavage, it is likely to remain bound to the ECM and does not diffuse into the exudate. Overall, BaP1 displays a higher ability to degrade not only BM components, i.e. type IV collagen, perlecan, and nidogen, but also type VI and XV collagens, which play a role in the integration of BMs with their surrounding matrix. Another extracellular matrix protein present in higher amounts in exudate collected from mice injected with BaP1 is thrombospondin 1. This may be a consequence of platelet aggregation secondary to microvessel damage and hemorrhage [72]. This counteradhesive protein, in turn, may contribute to an increase in vascular permeability and in the separation of cells from the matrix, owing to their capacity to inhibit cell-matrix and cell-cell interactions [72].

The structural basis for the functional differences described remains unknown, since BaP1 and leuc-a have similar structures. Analysis of the peptide cleavage consensus sequences on a proteome-derived library, by using mass spectrometry, revealed that these enzymes have a clear preference for leucine in the P1′ site. However, leuc-a has a preference for aspartate in the P4′ site, whereas BaP1 has a preference for alanine in this position [42]. Thus, differences in the consensus sequences for the P4-P4′ sites between SVMPs may bear functional consequences related to their ability to hydrolyze BM substrates, an issue that requires further investigation. In addition, variations in protein backbone flexibility of a loop located close to the active site of these enzymes have been proposed to play a role in the hemorrhagic activity of P-I SVMPs [34]. Further studies with a larger number of hemorrhagic and non-hemorrhagic P-I SVMPs are necessary to test these hypotheses and to discern the structural basis behind the highly variable hemorrhagic potential of P-I SVMPs.

In conclusion, the present study provides clues to understand the differences between the action of hemorrhagic and non-hemorrhagic P-I SVMPs *in vitro* and *in vivo,* and offers insights into the role of various ECM components in microvessel stability. BaP1 and leuc-a differ in their capacity to degrade key BM substrates, mainly type IV collagen, perlecan and, to a lesser extent, nidogen, as well as proteins which play a role in the integration of BMs with the surrounding matrix, i.e. type VI and type XV collagens. The drastic difference in type IV collagen hydrolysis between these enzymes strongly suggests that degradation of this structurally-relevant BM component is likely to represent a key step in the mechanism of action of hemorrhagic SVMPs, and underscores the key role played by this ECM protein in the mechanical stability of capillary blood vessels.
